# Combined Hip Procedure in a 74-Year-Old Patient with Acetabulum Fracture

**DOI:** 10.7759/cureus.62714

**Published:** 2024-06-19

**Authors:** Mohammed H Fallatah, Mohammed Sabr

**Affiliations:** 1 Department of Orthopedic Surgery, King Fahad General Hospital, Madinah, SAU; 2 Department of Orthopedic Surgery, Ministry of the National Guard - Health Affairs, Prince Mohammed Bin Abdulaziz Hospital, Al-Madinah, SAU

**Keywords:** geriatric hip fracture, total hip arthroplasty, combined hip procedure, elderly, acetabular fracture

## Abstract

In elderly individuals, low-energy trauma can cause acetabular fractures. Many surgical options have been described, including open reduction and internal fixation (ORIF), arthroplasty, and combined hip procedure. We present a 74-year-old man with a fall-related acetabular fracture with an unusual pattern, which was managed by ORIF and total hip arthroplasty. The patient experienced a hip dislocation at three weeks post-operation that was reduced in the emergency department. At 12 months post-operation, the patient resumed the same level of function he had before falling, using a cane while outside his home and sometimes inside. The combined hip procedure is associated with early weight bearing and excellent Harris hip scores, but proper preoperative planning and patient optimization are needed.

## Introduction

With the growing geriatric population, the incidence of hip fractures has increased. In the past three decades, there has been a 2.4-fold increase in the number of acetabular fractures in people older than 60 years [1]. The acetabular fracture can occur as a result of either high-energy trauma such as a car accident or low-energy trauma such as a simple fall, particularly in elderly people who have fragile bones [2,3]. Acetabular fractures with displacement of the anterior column are significantly more common in elderly patients than in younger patients [1]. Seventy percent of people over 65 with acetabular fracture had either an anterior column/posterior hemi-transverse fracture pattern or an associated both-column fracture pattern [4]. Operative and non-operative management have been designed to manage such fractures. The current report provides a detailed history of a 74-year-old man with an unusual acetabulum fracture and describes the findings and management of this case.

## Case presentation

A 74-year-old man presented to the hospital with left hip pain and an inability to bear weight on his left lower limb. The onset occurred four days earlier after he collapsed while standing in a restaurant. He fell on his left hip and lost consciousness for about 10 minutes. He had a similar attack of losing consciousness three months earlier without falling.

The patient’s pain was localized over his left hip. Pain started directly after the fall, it was sharp and progressive, increased with moving, and decreased with simple analgesia. This pain was associated with left hip and thigh swelling and an inability to bear weight on the left lower limb. The patient reported that the pain was severe enough to awaken him from sleep.

The patient’s medical history included hypertension, uncontrolled diabetes mellitus on medications, and a right acetabulum fracture managed by a total hip replacement six years prior to presentation. Since the surgery, he has been using a cane because of mild pain in the right hip. The patient’s pain was well controlled with simple analgesia.

On examination, the patient was conscious and alert, with stable vital signs. A surgical scar was present on his right hip from the previous operation, with no signs of infection or wound complications. His right hip, knee, and ankle joints each showed a good range of motion, and his distal neurovascular status was intact. The patient reported no calf muscle tenderness.

The patient’s left hip showed mild swelling, but no wounds or ecchymosis. The patient had tenderness over the anterior aspect, and the hip motion was painful and limited. He had full motion of the ankle and no calf muscle tenderness, and distal neurovascular status was intact.

**Figure 1:**
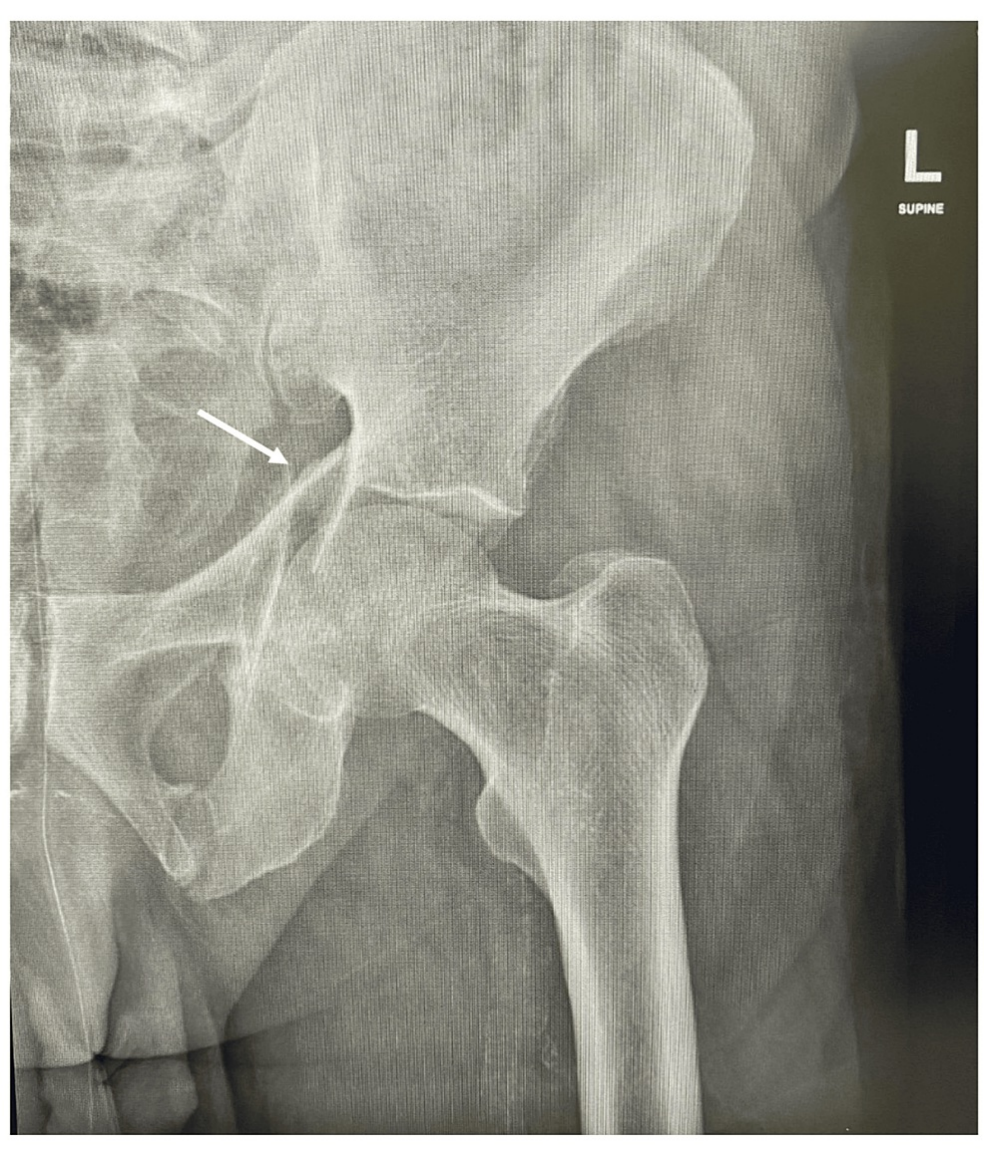
Anteroposterior (AP) x-ray of the left hip showing left acetabulum and inferior pubic ramus fracture.

**Figure 2:**
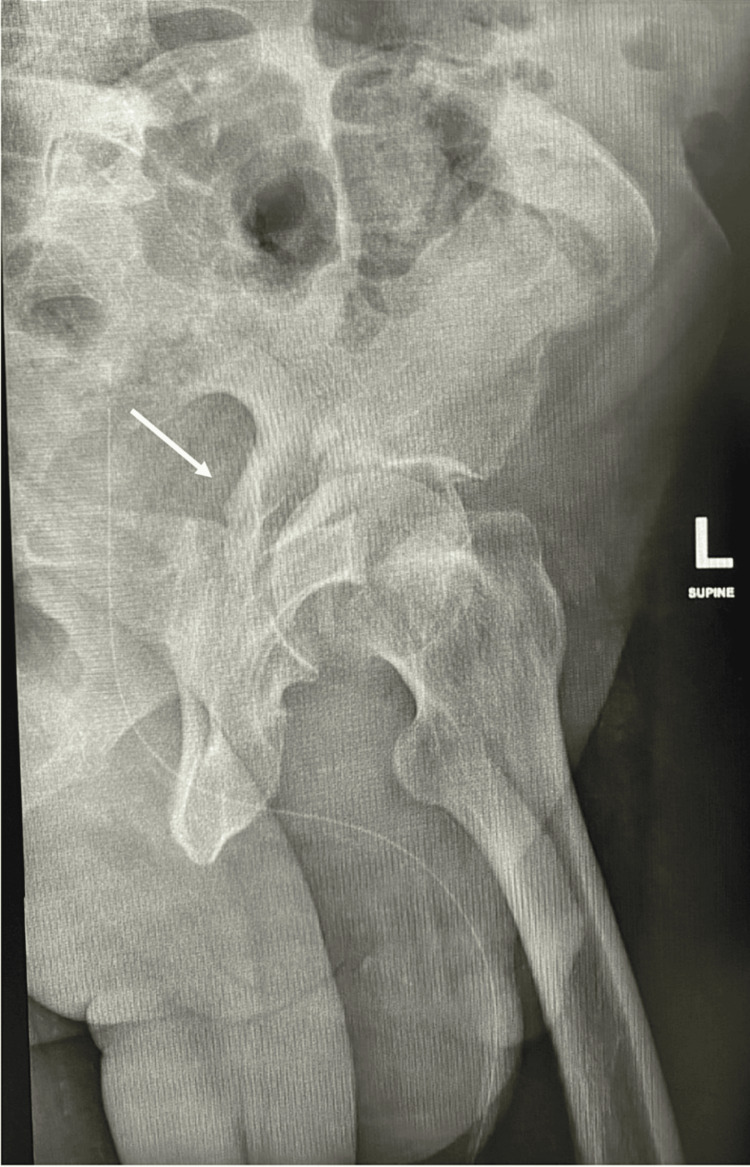
Iliac oblique view of the left hip showing posterior column fracture.

Laboratory investigations were done and included basic laboratory tests. Results showed high glucose and HbA1C levels, but the cardiac profile was within normal limits was reassuring. Doppler ultrasound was done for both limbs, and the results were negative for deep vein thrombosis. Electrocardiography and echocardiography were also done, with a cardiology review. Pelvic x-rays showed disruption of iliopectineal and ilioischial lines, with a fracture line through the obturator foramen (Figures 1, 2).

**Figure 3:**
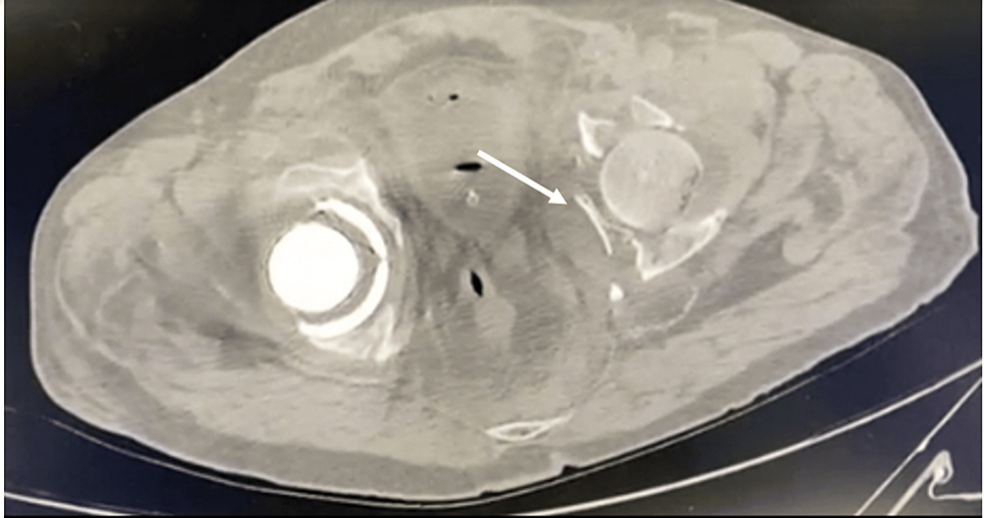
Axial cut CT scan of the pelvis showing left acetabulum fracture with displacement. CT: Computed tomography

**Figure 4:**
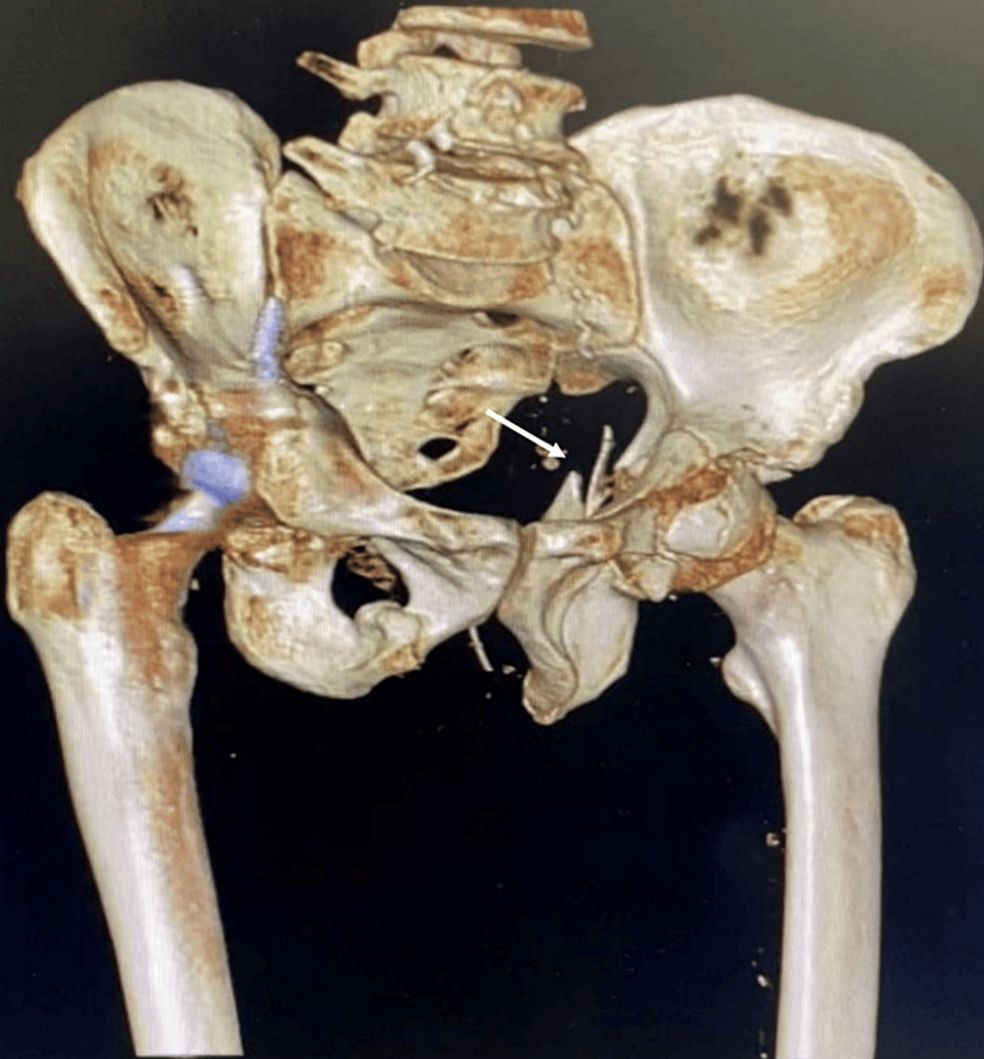
3D reconstruction of the pelvis showing left acetabular posterior column fracture.

**Figure 5:**
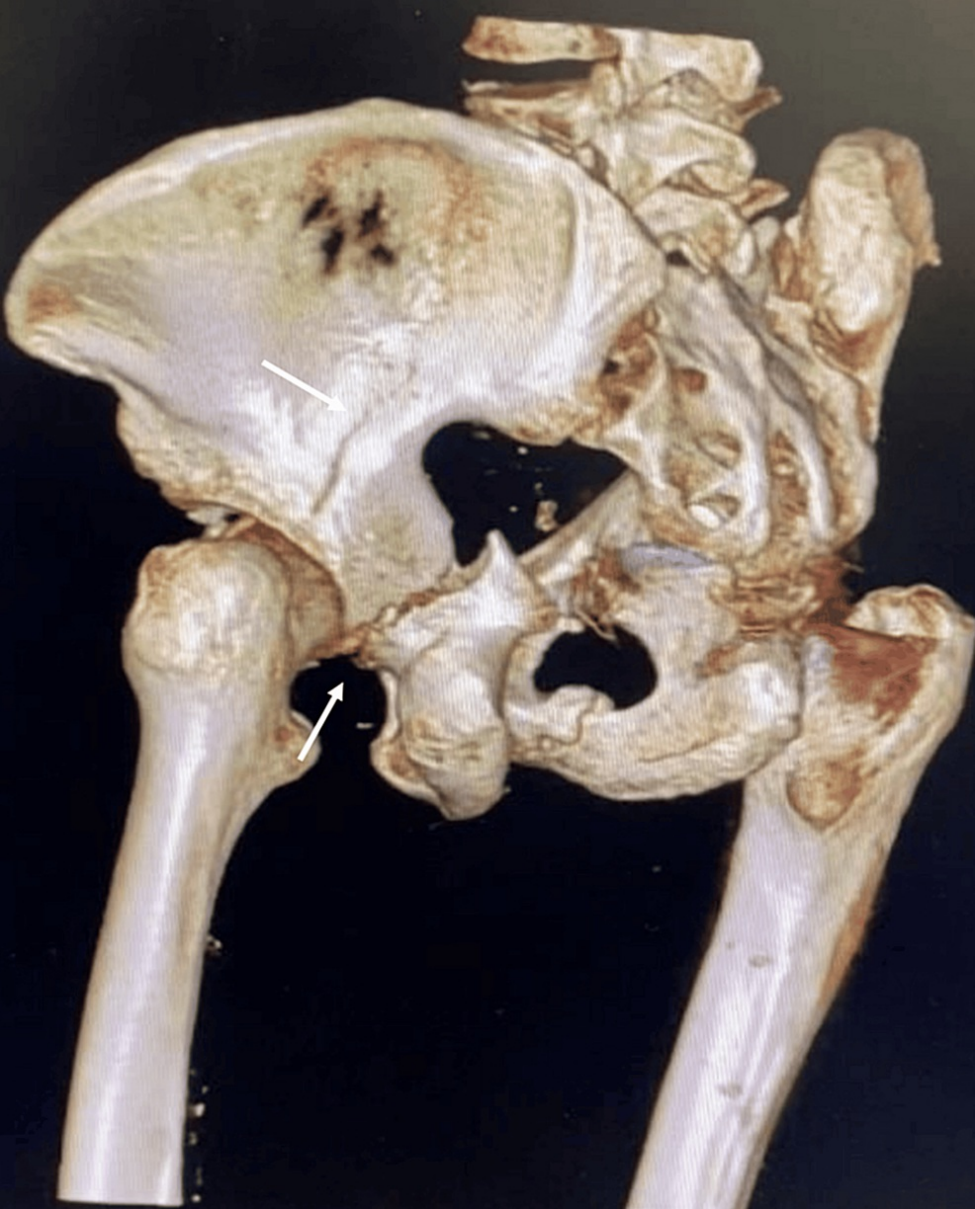
3D reconstruction of the pelvis showing fracture line extending from the iliac bone to the posterior column and posterior column fracture.

Computed tomography (CT) of the pelvis with three-dimensional reconstruction was done to characterize the fracture and for preoperative planning. The scan showed a T-shaped acetabulum fracture with another fracture extending from the iliac bone to the posterior column (Figures 3-5).

Open reduction and internal fixation (ORIF) of posterior column fracture through a posterior approach were planned, with total hip arthroplasty (THA) in the same surgery. The surgery was done by two senior consultants, including a trauma surgeon and an arthroplasty surgeon.

**Figure 6:**
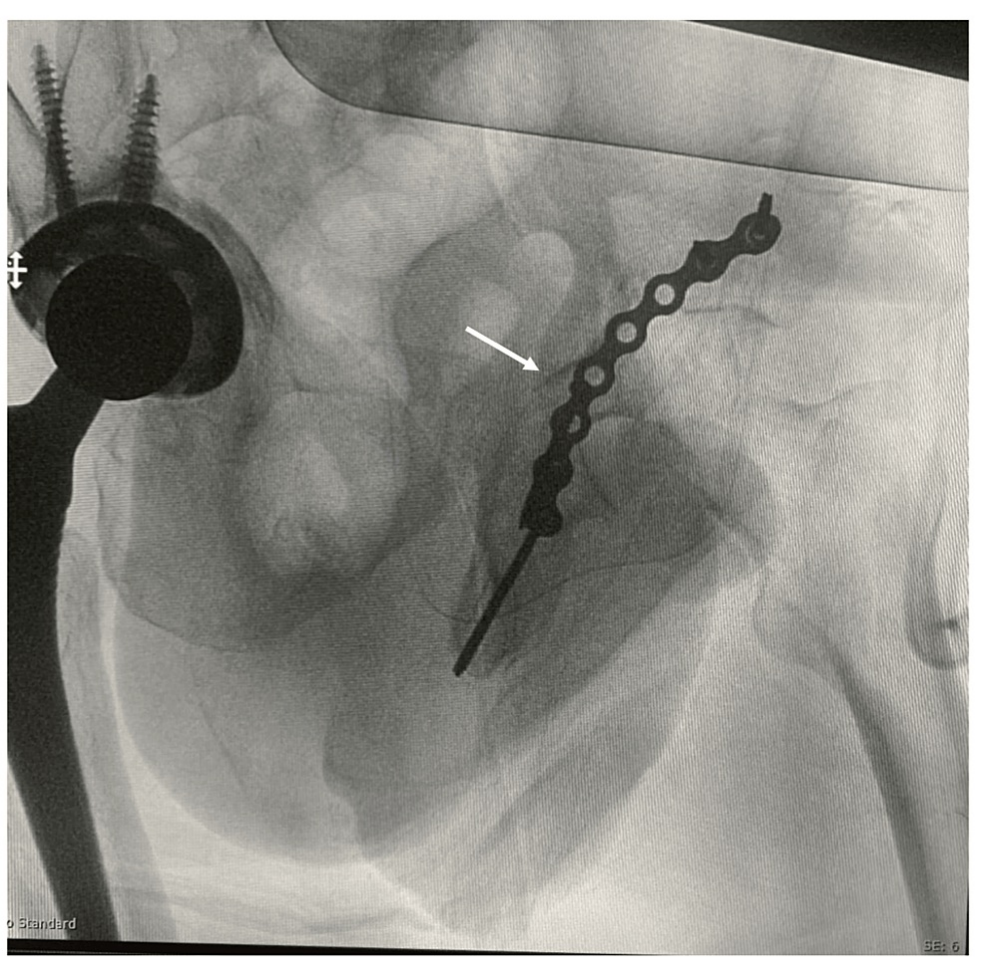
Intra-operative iliac oblique view x-ray showing fixation of the posterior column with plate and screws.

**Figure 7:**
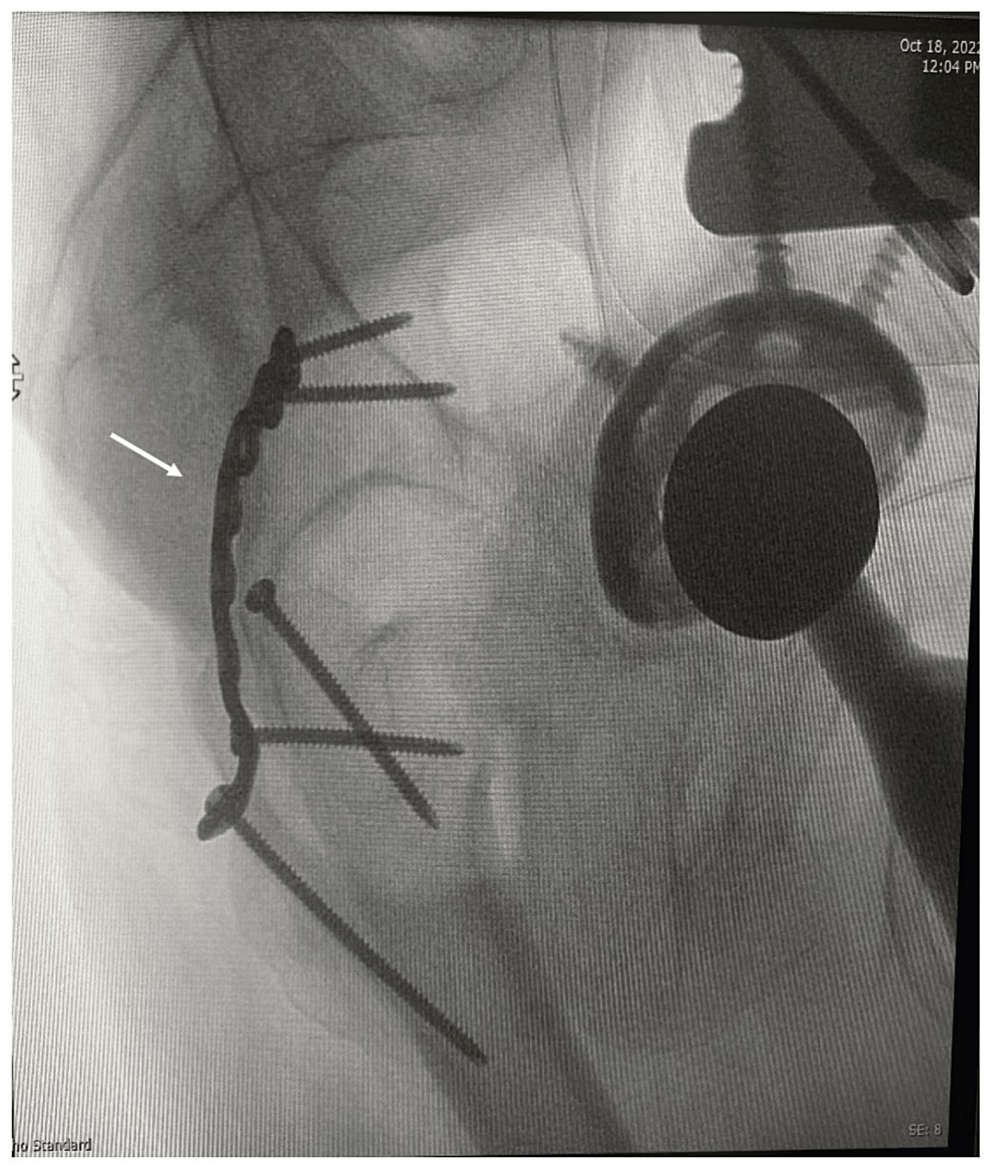
Intra-operative lateral pelvis x-ray showing fixation of the posterior column with plate and screws.

Prior to surgery, good control of blood pressure and glucose level was achieved, and cardiology review and clearance were obtained. The patient received general anesthesia and was placed in the right lateral decubitus position. A Kocher-Langenbeck approach was used. The short external rotator was exposed, and the piriformis muscle was identified and cut at its insertion in the greater trochanter. The sciatic nerve was identified and protected. Exposure of the ilium was completed by reflection of the gluteus muscle. The iliac wing fracture extending down to the posterior column was identified, and another fracture line in the inferior part of the posterior column was also identified as part of the T-shaped fracture. Surgical dislocation was done, and the head and neck were removed using a saw. Posterior column fractures were reduced and fixed with lag screws and a plate. As we used the posterior approach, the anterior column fracture could not be reached (Figures 6, 7).

**Figure 8:**
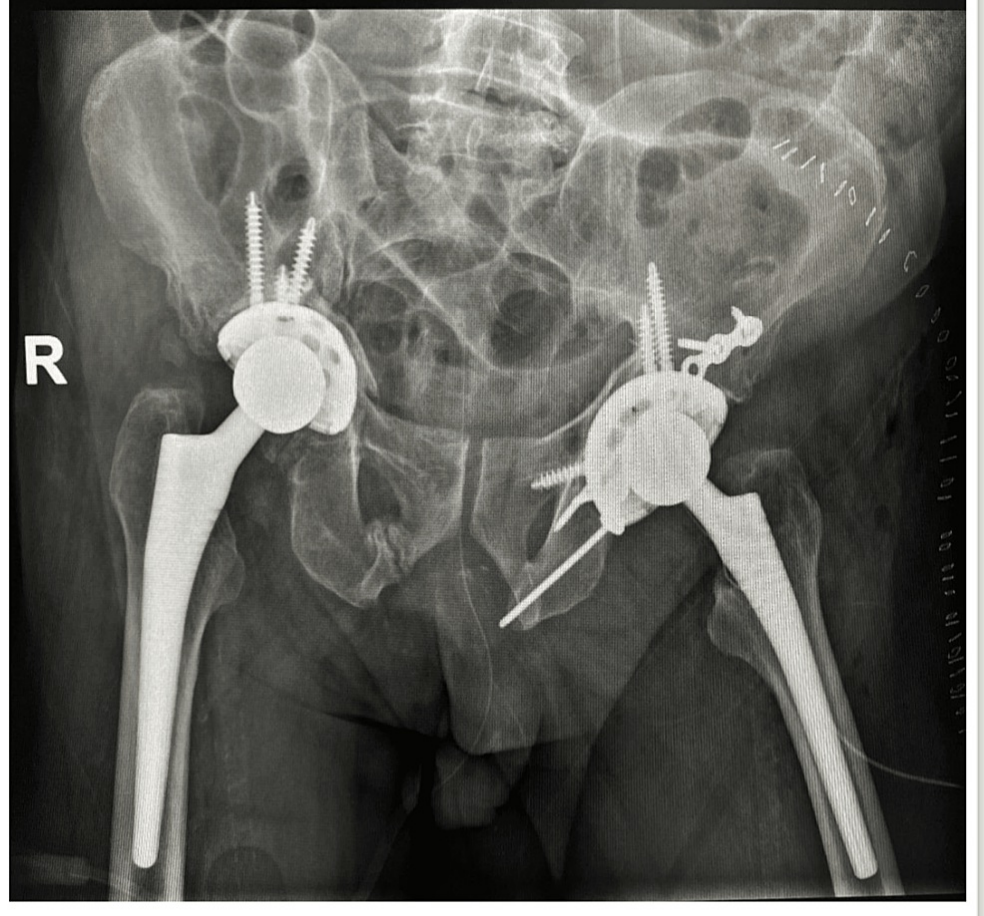
Post-operative x-ray of the pelvis showing left hip ORIF and THA. ORIF: Open Reduction Internal Fixation, THA: Total Hip Arthroplasty.

**Figure 9:**
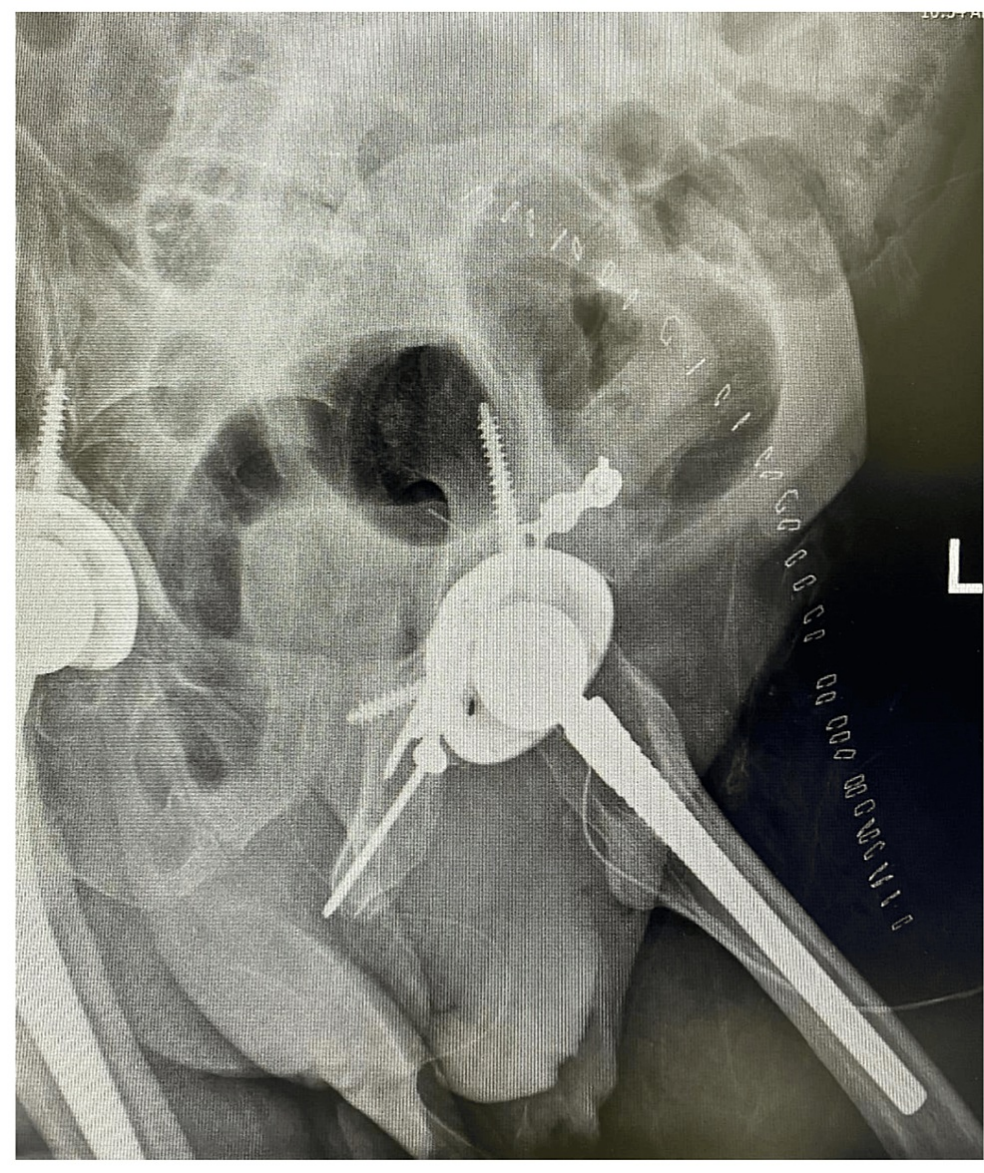
Post-operative obturator oblique view of the left hip.

Reaming of the acetabulum was carried out to size 57 mm, and a 60-mm hydroxyapatite-coated cup with multiple holes was inserted. A polyethylene liner was inserted in the cup, and we were able to put four screws through the cup, one into the ischium and the others into the ilium. For the femur, we used a cementless, tapered stem that was fully coated with hydroxyapatite and a 32-mm femoral head. Reduction of the left hip was done, and it was stable in 90° flexion and internal rotation up to 45°. Vancomycin powder, tranexamic acid, and bupivacaine were applied to the hip. The patient tolerated the surgery well, and he was planned for bed-to-chair mobilization and to remain non-weight bearing till postoperative CT was done. Afterward, the patient was allowed partial weight bearing with a walker, and hip dislocation protective measures were taken (Figures 8, 9).

During his hospital stay, the patient developed aspiration pneumonia, which was managed with antibiotics. He also developed upper gastrointestinal bleeding, and endoscopy was done with gastroenterology. Esophagitis was diagnosed, and the patient started an omeprazole infusion. Both complications were successfully treated, and the patient was discharged to go home on postoperative day 8 in good condition.

At 21 days after surgery, the patient presented at the clinic with deformity, shortening of the left lower limb, and an inability to bear weight on the left lower limb. An x-ray was done and showed left hip dislocation. Reduction was done in the emergency department and skin traction was applied, and the patient was admitted for two days for observation. He was then again discharged in good condition.

**Figure 10:**
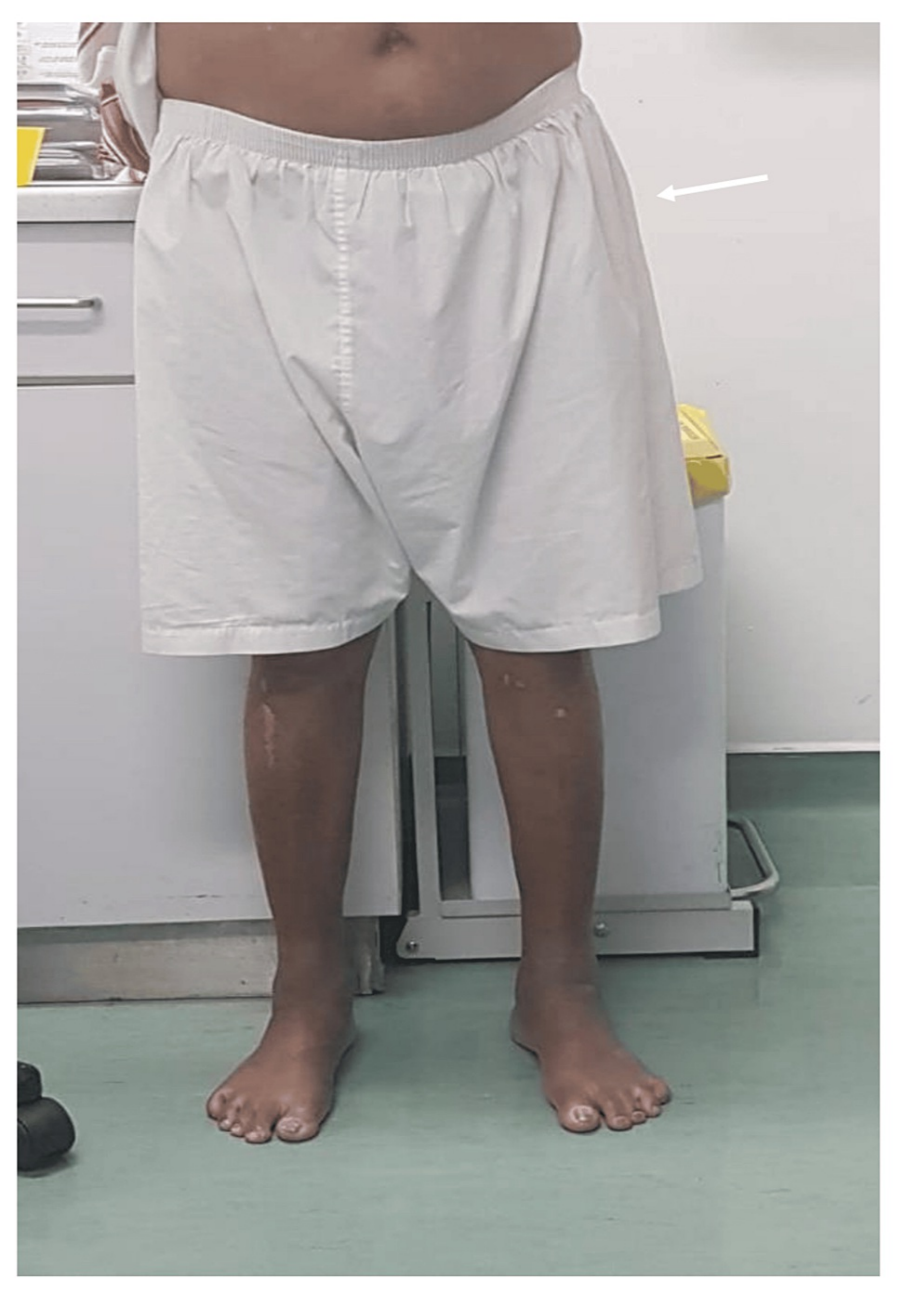
Post-operative clinical picture showing patient standing without assistance.

**Figure 11:**
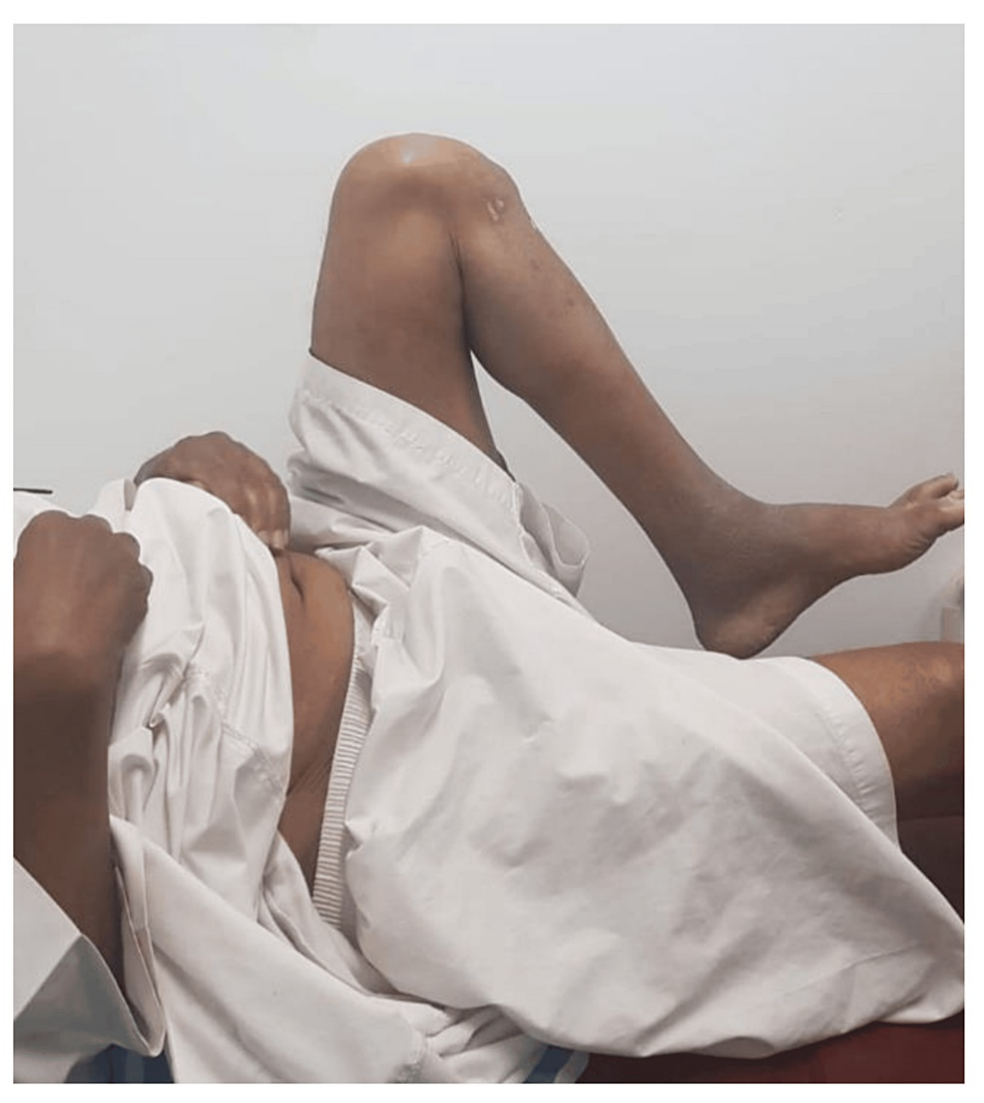
Post-operative clinical picture showing left hip flexion.

The patient was seen in the clinic at 30, 44, 52, and 65 days after surgery. The wound was healed with no pus discharge, and no other episodes of dislocation occurred. The patient used a walker in the house and a wheelchair outside the house. At three months after surgery, the patient started quadriceps strengthening and then progressed to full weight bearing. At six and 12 months, the patient resumed the same level of function as before the trauma, when he used a cane outside his house and sometimes inside his house (Figures 10, 11).

## Discussion

The incidence of acetabular fracture is increasing as the geriatric population grows [1]. The one-year mortality rate associated with acetabular fracture is 8%-16% [5,6], which is less than that with proximal femur fracture [7]. The mortality rate in elderly patients with an isolated acetabular fracture is less than that in elderly with polytrauma including acetabulum fracture [5]. The most common mechanism of acetabular fracture in elderly people is low-energy trauma [8,9]. Such fractures can lead to serious articular destruction, as in our case.

Our patient had a T-shaped acetabular fracture, with another fracture line extending from the iliac bone to the posterior column. This fracture pattern was unusual, as the most common fracture patterns in the elderly are anterior column posterior hemi-transverse and associated both-column fractures [1].

As most elderly patients present with comorbidities, good assessment, and preoperative optimization are required. Our patient was assessed by medical, cardiology, and anesthesia teams.

Several treatment strategies for acetabulum fractures in the elderly have been documented in the literature. A non-surgical option is reserved for fractures with minor displacement and a low risk of post-traumatic arthritis (PTA). Surgical options including ORIF, THA, or a combined procedure are reserved for patients with displaced fractures and PTA risk [10,11]. We chose a combined hip procedure (CHP) as the patient is elderly and has a comminuted, displaced acetabulum fracture.

Rickman et al. [12] found that using CHP including trabecular metal allows immediate full weight bearing with very few complications. In a study by Salama et al. [13], patients started toe-touch weight bearing for six weeks and then progressed to full weight bearing at that point. The authors found that that all patients who underwent CHP could walk independently; 72.7% had excellent Harris hip scores and 27.7% had good results. Our patient started partial weight bearing after a postoperative CT scan, and he walked without a cane inside the house for six months.

Regarding complications, Capone et al. [14] conducted a systemic review that included 354 patients with acetabular fractures that were managed by either ORIF or arthroplasty (THA/CHP). They found that ORIF for acetabular fracture had a longer operating time, but less blood loss, than CHP. Heterotopic ossification and deep infection were observed with both procedures. The rate of hip dislocation was 4.8% in THA-only patients and 8.9% in CHP patients [14]. Our patient experienced a posterior hip dislocation 21 days after surgery. Borg et al. [15] confirmed that CHP confers a considerably reduced need for further surgery compared with ORIF alone in elderly patients with complex acetabular fractures. In addition, they reported that the mortality rate is lower with CHP than ORIF [15].

In their literature review, Delgadillo et al. [16] found that CHP, or “fix-and-replace technique,” was associated with decreased mortality and morbidity. However, adequate preoperative planning and reconstruction were needed to meet early rehabilitation requirements [16].

Little is known about the mid-term outcome of CHP. Ortega-Briones et al. [17] followed up with patients for four years and found no loosening or no late complications. Consequently, they considered CHP to be a viable long-term solution.

## Conclusions

Low-energy trauma can cause acetabular fractures in elderly individuals. Many surgical options have been described, including ORIF, arthroplasty, and CHP. As most of the elderly patients present with comorbidities, good assessment, and preoperative optimization are required. With good patient selection and optimization, CHP done by expert surgeons yields excellent outcomes. It allows early weight bearing, limiting the need for further surgeries in the future and decreasing mortality and morbidity rates. The dislocation rate is more than pure THA. However, little is known about midterm and long-term outcomes, and further research is needed.

## References

[1] Ferguson TA, Patel R, Bhandari M, Matta JM (2010). Fractures of the acetabulum in patients aged 60 years and older: an epidemiological and radiological study. J Bone Joint Surg Br.

[2] Kannus P, Parkkari J, Sievänen H, Heinonen A, Vuori I, Järvinen M (1996). Epidemiology of hip fractures. Bone.

[3] Löfman O, Berglund K, Larsson L, Toss G (2002). Changes in hip fracture epidemiology: redistribution between ages, genders and fracture types. Osteoporos Int.

[4] Firoozabadi R, Cross WW, Krieg JC, Routt ML (2017). Acetabular fractures in the senior population—epidemiology, mortality and treatments. Arch Bone Jt Surg.

[5] Koval KJ, Zuckerman JD (1994). Functional recovery after fracture of the hip. J Bone Joint Surg Am.

[6] Gary JL, Paryavi E, Gibbons SD (2015). Effect of surgical treatment on mortality after acetabular fracture in the elderly: a multicenter study of 454 patients. J Orthop Trauma.

[7] Bible JE, Wegner A, McClure DJ, Kadakia RJ, Richards JE, Bauer JM, Mir HR (2014). One-year mortality after acetabular fractures in elderly patients presenting to a level-1 trauma center. J Orthop Trauma.

[8] Moed BR, WillsonCarr SE, Watson JT (2002). Results of operative treatment of fractures of the posterior wall of the acetabulum. J Bone Joint Surg Am.

[9] Negrin LL, Seligson D (2017). Results of 167 consecutive cases of acetabular fractures using the Kocher-Langenbeck approach: a case series. J Orthop Surg Res.

[10] Heimke IM, Scarcella NR, Simske NM, Furdock R, Vallier HA (2022). Surgical versus nonsurgical management of acetabular fractures with associated patterns in elderly patients: factors affecting outcomes. J Am Acad Orthop Surg Glob Res Rev.

[11] Soni A, Gupta R, Sen R (2022). Acetabulum fractures in elderly patients: a review. Chin J Traumatol.

[12] Rickman M, Young J, Bircher M, Pearce R, Hamilton M (2012). The management of complex acetabular fractures in the elderly with fracture fixation and primary total hip replacement. Eur J Trauma Emerg Surg.

[13] Salama W, Mousa S, Khalefa A, Sleem A, Kenawey M, Ravera L, Masse A (2017). Simultaneous open reduction and internal fixation and total hip arthroplasty for the treatment of osteoporotic acetabular fractures. Int Orthop.

[14] Capone A, Peri M, Mastio M (2017). Surgical treatment of acetabular fractures in the elderly: a systematic review of the results. EFORT Open Rev.

[15] Borg T, Hernefalk B, Hailer NP (2019). Acute total hip arthroplasty combined with internal fixation for displaced acetabular fractures in the elderly: a short-term comparison with internal fixation alone after a minimum of two years. Bone Joint J.

[16] Delgadillo C, Pesantez R (2023). Fix and replace technique in elderly acetabular fractures. J Musculoskeletal Surgery and Research.

[17] Ortega-Briones A, Smith S, Rickman M (2017). Acetabular fractures in the elderly: midterm outcomes of column stabilisation and primary arthroplasty. Biomed Res Int.

